# Inhibitory Control Predicts Grammatical Ability

**DOI:** 10.1371/journal.pone.0145030

**Published:** 2015-12-14

**Authors:** Paul Ibbotson, Jennifer Kearvell-White

**Affiliations:** Faculty for Education and Languages, Open University, Milton Keynes, MK7 6BJ, United Kingdom; Max Planck Institute for Human Cognitive and Brain Sciences, GERMANY

## Abstract

We present evidence that individual variation in grammatical ability can be predicted by individual variation in inhibitory control. We tested 81 5-year-olds using two classic tests from linguistics and psychology (Past Tense and the Stroop). Inhibitory control was a better predicator of grammatical ability than either vocabulary or age. Our explanation is that giving the correct response in both tests requires using a common cognitive capacity to inhibit unwanted competition. The implications are that understanding the developmental trajectory of language acquisition can benefit from integrating the developmental trajectory of non-linguistic faculties, such as executive control.

## Introduction

Innate mental representations of grammar were initially proposed because it was thought that general learning mechanisms were not powerful or subtle enough to explain adult language competence [1]. The original claim was made when ‘learning mechanisms’ more-or-less meant the associationist framework of Behaviourism. Decades of work since then has shown the child has a much richer and more sophisticated set of cognitive and social resources to bring to bear on the language acquisition process than the Behaviourist conception of learning [2,3,4,5,6,7,8]. Despite advances, much painstaking work still remains to be done to show exactly how aspects of cognition (e.g., memory, attention, executive function) interact with social reasoning (e.g., intention-reading, cultural intelligence) in a way that guides the child to an adult grammar. One branch of linguistics that has taken up this challenge is Cognitive Linguistics. It seeks to render accounts of language “consonant with aspects of cognition which are well documented or self-evident, or at least highly plausible, and which may well be manifested in non-linguistic activities” ([9] p.9). Contrast this with the Chomskian position which stresses language as separate from the rest of cognition: “It would surprising indeed if we were to find that the principles governing [linguistic] phenomena are operative in other cognitive systems…there is good reason to suppose that the functioning of the language faculty is guided by special principles specific to this domain …” ([10] p. 44).

Here we take forward the Cognitive Linguistics enterprise and explore a specific conjecture about the relationship between language and cognition. The hypothesis is this: grammatical ability to produce an irregular past tense form, for example, *fly* → *flew*, depends on the ability to inhibit a temping but incorrect response, *flyed*. The idea is that the correct form *flew* is facing unwanted competition from analogous patterns such as *tie* → *tied*, *die* → *died* and *lie* → *lied*. So, to put it very simply, if children are to learn language they must learn patterns and they must learn exceptions to those patterns. Giving the correct linguistic response involves suppressing this competition by using a cognitive faculty that is independent of language—inhibition.

The prediction is that those participants who are good at avoiding the overgeneralisation error (e.g., *flyed*) should also be good at inhibition. The implications of this are (1) it provides evidence that performance on a linguistic and non-linguistic test are recruiting the common cognitive faculty of inhibition, strengthening the case that language is deeply integrated with the rest of cognition (2) it provides new insights into the process of language acquisition. These overgeneralisation errors have traditionally received a very linguistic, domain-internal analysis [11,12,13,14,15]. A positive result would open the door to more cognitive-based explanations of the phenomena, for example, the retreat from overgeneralisation errors witnessed in child development could be the result of maturing inhibitory control (3) it identifies a source of individual variation in language ability which may in turn have implications for linguistic interventions, particularly for those at the far end of the spectrum of language ability like those with Specific Language Impairment.

As a test of grammatical ability we use a past tense elicitation task where participants hear a standard frame “…*every day I fly*, *yesterday I*….” and have to complete the sentence. As a test of inhibition we use the Sun-Moon Stroop task which involves participants responding “*sun*” to a picture of a moon and vice versa. There is evidence to suggest bilinguals are better than monolinguals at Stroop tests as they are well practiced in the skills of cognitive control and conflict resolution which switching between languages requires [16,17] (but also see [18]). Here we test whether the variation within monolinguals is also related to their ability to inhibit. To test this we use 5-year-old participants because adult monolinguals are at ceiling performance on the past tense elicitation task, meaning that there would be no between-participant variation to investigate. It is worth noting however, that these errors are not entirely absent from adult speakers, especially when the speaker is tired or under some communicative stress suggesting these too could be elicited under different experimental conditions than the ones we use here.

**Fig 1:**
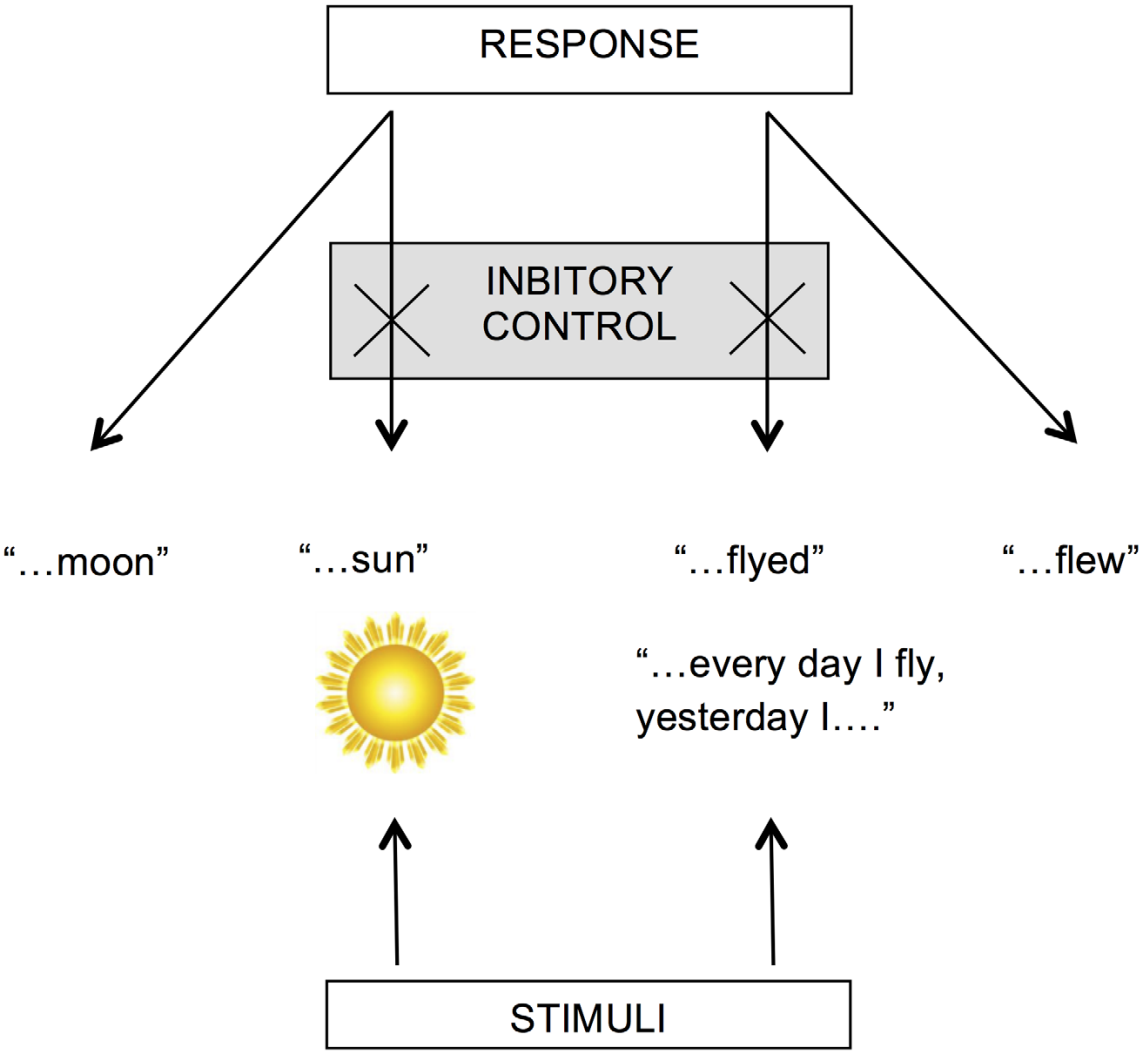
Hypothesised relationship between performance on the non-linguistic test (Stroop) and the linguistic test (past tense elicitation). Giving the correct linguistic response involves suppressing competitors by using a common cognitive faculty—inhibition.

It could be that those children who are good at the Stroop test simply know more words or are a little older and this makes them better at the grammatical task. For this reason we also recorded each participant’s vocabulary ability and age in months. All three variables will be assessed to see what extent they predict grammatical ability. The main hypothesis is schematically summarised in Fig 1.

## Method

### Participants

A total of 81 five-year-olds participated in this study (*M* = 5;6, *SD* = 3.54), 44 male, 37 female. All children were recruited from schools located in the Milton Keynes, Market Harborough and Henlow areas of the UK. All research conducted adhered to the British Psychological Society code of Ethics. The research was approved by the Open University Ethics Board. The purpose of the experiment was communicated to parents who chose to opt-in their children by a consent letter. Children were free to discontinue the experiment by assent.

### Materials and procedures

Each child completed the Past Tense Task-20 [19], the English Peabody Picture Vocabulary Test [20] and the Sun-Moon Stroop test [21]. All tasks were presented individually to each child in a quiet room of their school. The presentation of the tasks was counterbalanced.

### Past Tense Elicitation Task

The PPT-20 was developed by Conti-Ramsden et al [19] as an adaption of the well-known Marchman et al [22] past tense task. This version is shorter, provides appropriate UK norms and is easily administered to children of this age group. For each item, children are presented with a set of three consecutive sentences, with an accompanying picture depicting a scene where an action is taking place. The third sentence has a missing word at the end and the child is asked to fill in the gap to describe ‘what happened yesterday’. Rising intonation suggests that the sentence is incomplete. The sentences all follow the format:

*This boy is walking*. *He walks everyday*. *Yesterday he*…?

Three practice items are administered first (‘walk’, ‘fish’, ‘catch’), followed by the test items. When more than one response was provided (that is, self-corrections), coding was based on the final production. Both ‘spelled’ and ‘spelt’ were accepted as correct responses for the item containing the verb ‘spell’ as both forms are generated with equal frequency by speakers of British English. Although the PPT-20 records various types of error (e.g., non responses, responses with non-target verbs, no change of verb stem) we were interested in suffixation errors which comprised the addition of a stem-final suffix of vowel change plus suffix, for example *fly* → *flyed*. This is because our hypothesis is concerned with inhibiting the competitors that this type of overgeneralisation involves. A list of verbs used in this study appears in S1 File.

### Sun-Moon Stroop

Following the procedure of Archibald and Kerns [21], the Sun—Moon Stroop consists of two conditions. In condition A, children are shown a single page consisting of 30 sun and moon pictures, which are randomly arranged into equal rows and columns. Children are then instructed to say ‘sun’ to the pictures of the suns, and ‘moon’ to the pictures of the moons, as fast as they can (within a 45-s time limit) and to correct themselves if they made a mistake before moving on. The experimenter (E) points to each picture as it is named, and moves across the rows. If a child makes an error on a picture, E leaves his finger on this picture until the self-correction. Children are told that if they name all the pictures on the page within the given time limit, they start from the top again. As a practice trial, children are asked to name the first four pictures. In condition B, the stimuli were arranged differently but consisted of the same number of pictures. Children are now instructed to say ‘moon’ to a picture of a sun, and ‘sun’ to a picture of a moon. We were specifically interested in the number of incorrect responses in Condition B because participants have to inhibit the tempting response to say “moon” to a picture of the moon. This measure gives us the closest analogy to the past tense task where participants are also scored on number of incorrect responses.

### BPVS

The English Peabody Picture Vocabulary Test [20] is a receptive vocabulary test in which children are shown an array of four coloured pictures. The experimenter reads out the target word to them, and they are required to select the picture that matches the target word. The test becomes progressively more difficult; once children fail eight items within one block (of 12 items) the test is terminated.

## Results

First we wanted to check whether our participant group was broadly in line with UK norms on grammatical ability and vocabulary (Table 1).

**Table 1:** Summary statistics for the grammar and vocabulary tests.

	PPT-20	BPVS
Standardised Test	*M* = 7.50, *SD* = 2.79	*M* = 100, *SD* = 14.1
Present Study	*M* = 6.33, *SD* = 2.63 (50^th^ percentile or closest = 8 Normal Range Raw Score 16^th^ Percentile or closest above = ≥5)	*M* = 108.22, *SD* = 12.34 (standardised score based on mean age (5;6) of participants)

The mean scores for our participant were slightly lower than the average for the PPT-20 and slightly higher on the BPVS. Perhaps more importantly than the means, the standard deviations in our sample are broadly comparable to those of the wider population. This is important as it is this individual variation we are trying to account for and shows the amount of variation we have to work with in our sample is representative of the variance in the population. We now turn to our main research question. Our hypothesis is that there should be a positive correlation between inhibition and grammatical performance. In order to test whether the Stroop score is a predictor of verb errors we fitted a series of mixed effects logistic regression models using the glmer function in the R package lme4 (R script in S1 File; Raw data used in this analysis in S2 File). The glmer function estimates the parameters of the model using a maximum likelihood method.

In the models, the binary response variable denotes the presence or absence of a verb error. There are two grouping variables that we considered as random effects: participant ID (since each participating child has multiple responses) and verb (since each verb has a response from every participant). The fixed effects we considered were Stroop score, age and vocabulary score. The age and vocabulary variables are on a scale that is very different to Stroop score so, in order to help with the numerical stability of the model-fitting algorithm, these variables were each normalised by subtracting their mean and dividing by their standard deviation.

In order to determine the best form for the model, we began by fitting the most complex model we believed could be supported by the data. This model has all three main effects, random participant and verb intercepts, and random verb slopes for Stroop score, age and vocabulary score. We could not include random participant slopes because there is no variation of Stroop score, age or vocabulary score within participants. Nor could we include interactions between the main effects because including them caused the fitting algorithm to fail to converge, indicating that the sample size is too small to support this level of complexity. In R notation, the full model (Model 1) is:

Model 1:error ~ (1|participant)+(1+stroop+age+vocab|verb)+stroop+age+vocab

After fitting the full model we performed backwards selection on the random effects whilst keeping the fixed effects structure constant. We used AIC as the criterion for selecting terms to drop out of the model. The resulting model (Model 2) has random participant and verb intercepts and a random verb slope for Stroop score. In R notation Model 2 is:

Model 2:error ~ (1|participant)+(1+stroop|verb)+stroop+age+vocab

The reduced complexity of the random effects structure in Model 2 allowed us to then consider interactions between the main effects without running into convergence issues in the fitting algorithm. We therefore supplemented Model 2 with all two-way interactions between the main fixed effects and then performed backwards selection on the fixed effects whilst holding the random effects structure constant. Again, we used AIC as the criterion for selecting terms to drop out of the model. The backwards selection dropped age, vocabulary score and all interactions from the model, so the final model (Model 3) retains just the Stroop score as a fixed effect. In R notation Model 3 is:

Model 3:error ~ (1|participant)+(1+stroop|verb)+stroop

We took Model 3 as our final and best model in the analysis. The fact that Stroop score was retained as a fixed effect in the final model indicates that it is a predictor of verb errors. We went further and used the ANOVA function in R to perform a formal likelihood ratio test between Models 3 and 4, a reduced version that excludes Stroop score as a fixed effect. In R notation Model 4 is:

Model 4:error ~ (1|participant)+(1+stroop|verb)

The p-value for the likelihood ratio test is 0.021, indicating that the Stroop score effect is statistically significant at the 5% level.

Table 2 gives the estimated fixed effects coefficients from Model 3 along with their standard errors and 95% confidence intervals. In a logistic regression model, the exponential of the estimated coefficient for a numerical predictor such as Stroop score gives an estimate of the multiplicative effect on the odds of the outcome of a unit increase in the predictor. This means that a unit increase in Stroop score is associated with the odds of a verb error increasing by a factor of 1.25 with a 95% confidence interval given by (1.04, 1.55). This result is an average over participants and verbs. To give an example, the estimated error probability for an average verb in an average participant with Stroop score 5 is 0.76, compared to 0.80 for an average participant with Stroop score 6.

**Table 2:** Estimated coefficients and standard errors for the fixed effects in Model 3.

	Estimate	Standard error	Lower 95% confidence limit	Upper 95% confidence limit
**Intercept**	0.047	0.311	-0.595	0.686
**Stroop score**	0.225	0.095	0.037	0.436

In summary, Stroop score is found to be a statistically significant predictor of verb errors. A unit increase in Stroop score is associated with the odds of a verb error increasing by a factor of 1.25 with a 95% confidence interval given by (1.04, 1.55).

## Discussion

We have found evidence here that individual variation in grammatical ability can be predicted by individual variation in inhibitory control and perhaps it does so in way schematically shown in Fig 1. From other work we know grammatical categories such as the transitive can behave in similar ways to non-linguistic categories [23]. We also know speakers can use the eye-gaze of the speaker to work out the meaning of novel verbs [24] and grammatical constructions [25]. We perhaps can add past tense formation to that list of core linguistic abilities that are integrated at deep level with the rest of cognition. If the developmental trajectory of grammatical ability is in some sense dependent on the developmental trajectory of inhibition then the question is whether it makes sense to study these topics independently. For some phenomena it might not be productive to draw a sharp line between what is language and what is cognition. This is not, as Chomsky might respond, a “non-existence” argument—the idea that language does not exist as a serious independent topic of study. Rather, it emphasises the need to look at how deeply and complexly different areas of the mind and brain are integrated if we hope to explain these developmental trajectories. This integrated view of language and cognition is predicted by an evolutionary perspective on the development of language. For instance, language developed in our species perhaps as recently as 2–300,000 years ago. It is very likely then that language must have used and continues to use pre-existing cognitive mechanisms. As Evans and Levinson put it “The null hypothesis here is that all needed brain mechanisms, outside the vocal-tract adaptation for speech, were co-opted from pre-existing adaptations not specific to language” ([26] p. 447).

These results also bear on the processes of language acquisition. Overgeneralisation errors have received a lot of attention from developmental psycholinguists as they are thought to be a window in the processes that underlie linguistic creativity: Adults do not say ‘flyed’ so children must have created that form for themselves. One particular concern is how children ‘retreat’ from these errors if they never receive explicit feedback that they are incorrect. Currently, the three main solutions to this problem—pre-emption, entrenchment and semantic class—are all born out of a very linguistic analysis. Our result does not negate the need for these explanations (which offer more fine grained predictions than inhibition could) but it does suggest they should be considered along side more cognitive based explanations. We know inhibitory control is maturing throughout the years that children’s overgeneralisation errors are reducing [27]. It is possible therefore that inhibitory control accounts for some of the individual variation in past tense performance that linguistic accounts do not (and vice versa).

Finally, we know children with SLI struggle with the grammar of past tense formation [28], almost by definition. We also know that children with SLI have impaired performance on tasks involving inhibition [29]. Our result suggests further research on interventions designed to target both linguistic and non-linguistic components, with the hope that by raising the performance of one domain it might have a positive effect on the other.

The measure of inhibition we used in this study did involve participants recruiting language—they had to say the word ‘moon’ or ‘sun’. The main point is that ‘sun’ and ‘moon’ do not carry with them any prepotent linguistic response other than the one primed by the visual stimuli (pictures of suns and moons). This is a different situation to grammatical test where the tempting generalisation is generated by the linguistic system itself (analogy to other past tense forms). On reflection, an age appropriate non-verbal measure of Stroop would have been a cleaner measure and it would be interesting to see whether using such a test replicates our findings.

In summary, grammatical errors involving past tense formation and errors of performance on the Stroop test are significantly related. What appears to be the most parsimonious explanation for this relationship is that giving the correct response in both tests requires using a common cognitive capacity to inhibit unwanted competition. That performance on a linguistic and non-linguistic test are recruiting a common cognitive faculty of inhibition, strengthens the case that the complexity of language emerges through the interaction of cognition and language use over time [2,3,4,5,6,7,8].

## Supporting Information

S1 File Verbs used in this study (Figure A). R script (Figure B).
(DOCX)

S2 File Raw data. (XLSX)
