# Supplementary material for: Inhibitory Control Predicts Grammatical Ability
Source: PLoS One. 2015 Dec 14;10(12):e0145030. doi: 10.1371/journal.pone.0145030 (PMC4682623; doi:10.1371/journal.pone.0145030)
Supplement: S1 File — R script (Figure B). (DOCX) [file pone.0145030.s001.docx]

**Figure A**

Verbs used in this study.

| Item | Class |
| --- | --- |
| Draw | Irregular |
| Spell | Regular |
| Skate | Regular |
| Melt | Regular |
| Kiss | Regular |
| Drive | Irregular |
| Feed | Irregular |
| Fly | Irregular |
| Pick | Regular |
| Need | Regular |
| Mend | Regular |
| Bite | Irregular |
| Match | Regular |
| Taste | Regular |
| Try | Regular |
| Fall | Irregular |
| Send | Irregular |
| Hold | Irregular |
| Ride | Irregular |
| Sit | Irregular |

**Figure B**

R script.

The R output for the likelihood ratio test between Model 3 and Model 4 is as follows:

Models:

Model_4: error ~ (1 | participant) + (1 + stroop | verb)

Model_3: error ~ (1 | participant) + (1 + stroop | verb) + stroop

Df AIC BIC logLik deviance Chisq Chi Df Pr(>Chisq)

Model_4 5 980.59 1004.1 -485.29 970.59

Model_3 6 977.28 1005.5 -482.64 965.28 5.3062 1 0.02125 *

---

Signif. codes: 0 ‘***’ 0.001 ‘**’ 0.01 ‘*’ 0.05 ‘.’ 0.1 ‘ ’ 1

The R output summarising Model 3 is as follows:

Generalized linear mixed model fit by maximum likelihood (Laplace Approximation) ['glmerMod']

Family: binomial ( logit )

Formula: error ~ (1 | participant) + (1 + stroop | verb) + stroop

Data: dat

AIC BIC logLik deviance df.resid

977.3 1005.5 -482.6 965.3 804

Scaled residuals:

Min 1Q Median 3Q Max

-3.8551 -0.8288 0.4281 0.6392 2.5262

Random effects:

Groups Name Variance Std.Dev. Corr

participant (Intercept) 0.84632 0.9200

verb (Intercept) 0.20210 0.4496

stroop 0.02979 0.1726 -0.24

Number of obs: 810, groups: participant, 81; verb, 10

Fixed effects:

Estimate Std. Error z value Pr(>|z|)

(Intercept) 0.04715 0.31050 0.152 0.8793

stroop 0.22451 0.09505 2.362 0.0182 *

---

Signif. codes: 0 ‘***’ 0.001 ‘**’ 0.01 ‘*’ 0.05 ‘.’ 0.1 ‘ ’ 1

Correlation of Fixed Effects:

(Intr)

stroop -0.698
